# Genes of cell-cell interactions, chemotherapy detoxification and apoptosis are induced during chemotherapy of acute myeloid leukemia

**DOI:** 10.1186/1471-2407-9-77

**Published:** 2009-03-05

**Authors:** Anne Margrete Øyan, Nina Ånensen, Trond Hellem Bø, Laila Stordrange, Inge Jonassen, Øystein Bruserud, Karl-Henning Kalland, Bjørn Tore Gjertsen

**Affiliations:** 1 The Gade Institute, University of Bergen, Bergen, Norway; 2 Department of Microbiology and Immunology, Haukeland University Hospital, Bergen, Norway; 3 Department of Informatics, University of Bergen, Bergen, Norway; 4 Computational Biology Unit, Bergen Center for Computational Science, University of Bergen, Bergen, Norway; 5 Institute of Medicine, Hematology Section, University of Bergen, Bergen, Norway; 6 Department of Medicine, Hematology Section, Haukeland University Hospital, Bergen, Norway

## Abstract

**Background:**

The molecular changes *in vivo *in acute myeloid leukemia cells early after start of conventional genotoxic chemotherapy are incompletely understood, and it is not known if early molecular modulations reflect clinical response.

**Methods:**

The gene expression was examined by whole genome 44 k oligo microarrays and 12 k cDNA microarrays in peripheral blood leukocytes collected from seven leukemia patients before treatment, 2–4 h and 18–24 h after start of chemotherapy and validated by real-time quantitative PCR. Statistically significantly upregulated genes were classified using gene ontology (GO) terms. Parallel samples were examined by flow cytometry for apoptosis by annexin V-binding and the expression of selected proteins were confirmed by immunoblotting.

**Results:**

Significant differential modulation of 151 genes were found at 4 h after start of induction therapy with cytarabine and anthracycline, including significant overexpression of 31 genes associated with p53 regulation. Within 4 h of chemotherapy the BCL2/BAX and BCL2/PUMA ratio were attenuated in proapoptotic direction. FLT3 mutations indicated that non-responders (5/7 patients, 8 versus 49 months survival) are characterized by a unique gene response profile before and at 4 h. At 18–24 h after chemotherapy, the gene expression of p53 target genes was attenuated, while genes involved in chemoresistance, cytarabine detoxification, chemokine networks and T cell receptor were prominent. No signs of apoptosis were observed in the collected cells, suggesting the treated patients as a physiological source of pre-apoptotic cells.

**Conclusion:**

Pre-apoptotic gene expression can be monitored within hours after start of chemotherapy in patients with acute myeloid leukemia, and may be useful in future determination of therapy responders. The low number of patients and the heterogeneity of acute myeloid leukemia limited the identification of gene expression predictive of therapy response. Therapy-induced gene expression reflects the complex biological processes involved in clinical cancer cell eradication and should be explored for future enhancement of therapy.

## Background

Therapy with anthracycline and cytarabine results in modulation of a wide range of proteins including massive p53 protein activation followed by cell cycle arrest and apoptosis [1-3]. The p53 transcription factor transactivates a wide range of pro-apoptotic genes [4,5] involved in cancer cell elimination, and an intact p53 gene seems essential for therapeutic response in AML [6]. Most of the molecular mechanisms behind chemotherapy are elucidated in experimental systems and do not reflect tissue responses and the complex cell-cell interactions that are present *in vivo *[7]. As increasing evidence is proposing tumor-host mechanisms as important for effective chemotherapy [8], there is an immediate need to investigate these issues *in vivo *in human cancer.

Clinical response to chemotherapy and karyotype analysis of AML cells provide prognostic information about risk for relapse [9]. Gene expression analysis may provide important prognostic information in the 50% of patients with standard risk for relapse due to normal karyotype [10,11]. Recent studies of mutations or signaling response in AML have also indicated potential for risk stratification [12-14]. All these studies are based on bulk cell analysis, and propose that analysis of patient cells under DNA damaging therapy may provide biological important information about the therapy response. Common for most previous studies of chemotherapy induced gene expression is the time of sample collection at 24 h and later after start of chemotherapy [15,16]. We hypothesize that earlier sampling and analysis of gene expression could provide us with information about therapy responses and early resistance mechanisms against intensive chemotherapy.

In the present work we used high-density oligonucleotide microarrays to monitor therapy-induced changes of gene expression of AML blasts in seven *de novo *AML patients before, at 2–4 h and at 18–24 h after start of intensive chemotherapy infusion. There was no detectable decline in viability in the sampled cells. Early gene expression was dominated by p53-associated genes, while the later gene expression was dominated by genes involved in cytarabine detoxification, chemoresistance and cell-cell interactions.

## Methods

### Preparation of AML blasts

The study was approved by the local Ethics Committee (Regional komité for medisinsk og helsefaglig forskningsetikk, Vest-Norge; REK Vest) affiliated with the University of Bergen, and samples were collected after signed written informed consent. During the time period 2001–2003 we consecutively collected peripheral blood AML blasts from 7 patients with WBC counts above 1.5 × 10^9^/L eligible for treatment with chemotherapy. TP53 mutations are in general infrequent in AML [6] as confirmed in our previously described patient material where only two of 39 patients comprised mutated TP53 [17]. This analysis included patients 4 and 7 (Table 1) who were wild type for TP53. The patients were treated with intravenous infusion of idarubicine (12 mg/m^2 ^during 30 min on days 1–3) and cytarabine (200 mg/m^2 ^daily as continuous infusion on days 1–7). Peripheral blood was collected by antecubital vein puncture. Cells were prepared by density gradient separation (Ficoll-Hypaque; NyCoMed, Oslo, Norway; specific density 1.077). The percentage of blasts exceeded 95% for all patients [1]. A total of 20–40 million cells were pelleted and resuspended in Trizol (Invitrogen Corp., Carlsbad, CA). Cryopreserved cells from patients 3 and 8 (Table 1) sampled at diagnosis were exposed to 1.6 μM or 8.0 μM daunorubicin. (Pfizer Inc., New York, USA) *in vitro *(4 hours, 37°C, 5% CO2, humidified atmosphere, in serumfree medium (Stemspan H3000, StemCell Technologies, Vancouver, BC, Canada). The control cells were cultured with vehicle (NaCl 0.9%) and all cells were sampled at the 4 hours time point, before the appearance of any morphological signs (bright field and Hoechst epifluorescence microscopy) of apoptosis. Patients 1, 2 and 3 have previously been analyzed for gene expression (cDNA array only) after 4 h and presented in reference [1]. The clinical and biological characteristics of the patients are presented in Additional File 1.

### Microarray analysis

The cDNA microarray Agilent Human clone 1_clonesetB (Agilent Technologies, Inc., Palo Alto, CA) containing 12,814 unique clones, sourced from Incyte's UniGene 1 and Human Drug Target DNA clone sets http://www.ncbi.nlm.nih.gov/projects/geo/ was used for partial validation. Poly(A)RNA purification, cRNA synthesis, aminoallyl cDNA synthesis and hybridization were performed as previously described [18]. The Agilent Human Whole Genome Oligo Microarray (Agilent Technologies, Inc., Palo Alto, CA) was used to analyze samples. RNA was isolated from AML blasts and purification of poly(A)RNA was performed as described previously [18]. Aminoallyl-U (aa-UTP from Ambion, Austin, TX) was incorporated into cRNA followed by cross-coupling of Cy5- and Cy3 by means of reactive Cy-NHS compounds (Amersham Biosciences AB, Uppsala, Sweden) in order to generate fluorochrome labeled targets for DNA microarray analysis. Stratagene Universal RNA was used for reference Cy3-aa-cRNA preparation (Stratagene, La Jolla, CA). The hybridization procedure was done according to the Agilent protocols, except for a more stringent wash (0.1 × SSC at 35°C for 10 minutes for the Oligo Array). The oligonucleotide microarrays were scanned (Agilent Scanner G2505B) and features automatically extracted using Agilent Feature Extraction v.7.5. Annotated microarray data were uploaded in the BASE database and formatted and exported to ArrayExpress at the European Bioinformatics Institute http://www.ebi.ac.uk/arrayexpress (Accession number: E-TABM-632)) in agreement with the MIAME guidelines.

### Analysis of microarray gene expression data

Agilent's background subtracted signals (gIsWellAboveBG; rIsWellAboveBG) were used to filter spots lower than background intensity in both channels and intra-array normalization was carried out using lowess [19]. Genes with more than 30% missing values were removed and the remaining missing values were estimated using LSimpute [20]. The samples were divided into three groups: before, early (2–4 hours) and late (18–24 hours) after start of treatment and the mRNA expression levels of the treated samples were compared to the untreated ones. The Rank product method [21] was used to identify genes consistently up- or down-regulated. Gene ontology (GO) analysis was applied for functional studies using iterative group analysis (iGA) [22] utilizing the Source database http://source.stanford.edu[23]. False discovery rates for GO-terms were estimated by performing 100 permutations.

In the *ex vivo *experiment cell samples were treated with different doses (0, 1.6, and 8 μM) of anthracycline (daunorubicin). The untreated sample was kept as a control. Subsequently, the mRNA expression levels of the untreated sample were compared to the treated. To study if there was a general trend of up- or downregulation of genes in the treated compared to the untreated sample, the difference in expression levels was calculated for each gene. The genes were ranked according to expression difference for each of the three comparisons, e.g. three different rankings of the genes were produced, and these rankings were analyzed together using the rank product test [21]. The genes were then ordered by *p*-value for up- and downregulation in the treated samples relative to the untreated sample. On top of the rank product derived gene lists, we performed an iterative group analysis using GO terms as groups [22].

The gene expression profile of patients with FLT3 length mutation/internal tandem mutation (ITD) (2/7) was analyzed against patients harboring FLT3 wildtype (5/7) using Significant Analysis of Microarray (SAM) and Analysis of Variance (ANOVA) performed in J-Express http://www.molmine.com[24].

### Gene expression data validation using real time quantitative PCR

To confirm the observed differential gene expressions, we also performed qPCR by using TaqMan Low Density Arrays (T-LDA) (Applied Biosystems). We validated 22 upregulated genes and 8 downregulated genes of AML blasts sampled at start and at 4 h after start of treatment. The results of the T-LDA expression analysis were concordant with the Agilent cDNA and the oligo DNA microarray analyses (Additional File 2). The high reliability is reflected by correlation coefficients that varied from 0.72 to 0.96 between high density oligonucleotide microarrays and T-LDA values. The gene expression ratios of BCL2/BAX and BCL2/BCC3 were normalized, and p-value determined by a two-tailed Students t-test.

### Measurement of apoptosis

Apoptosis was determined in parallel samples for dual color flow cytometry with Annexin-V FITC and propidium iodide (APOTEST-FITC, Nexin Research, Kattendijke, The Netherlands), as described in Abrahamsen et al. 2002 [25]. Cell size was estimated in a hematology analyzer Abbott CELL-DYN 4000, and nuclear morphology was examined with epifluorescence microscopy analysis after DNA specific staining with bisbenzimide 33342.

### Protein purification, gel electrophoresis and immunoblotting

Material for protein analysis was collected according to standard procedures [1]. The p53 protein was detected using Bp53-12 monoclonal antibody, BAX protein was detected using primary 2D2 antibody, BCL-2 protein was detected using primary ΔC21 antibody and procaspase-3 protein was detected using primary E-8 antibody (Santa Cruz Biotechnology, Santa Cruz, CA) and actin protein was detected using primary AC-15 antibody (Abcam, Cambridge, UK).

## Results

### Pre-apoptotic AML cells in peripheral circulation were without signs of apoptosis during the first 24 hours of chemotherapy

**Figure 1:**
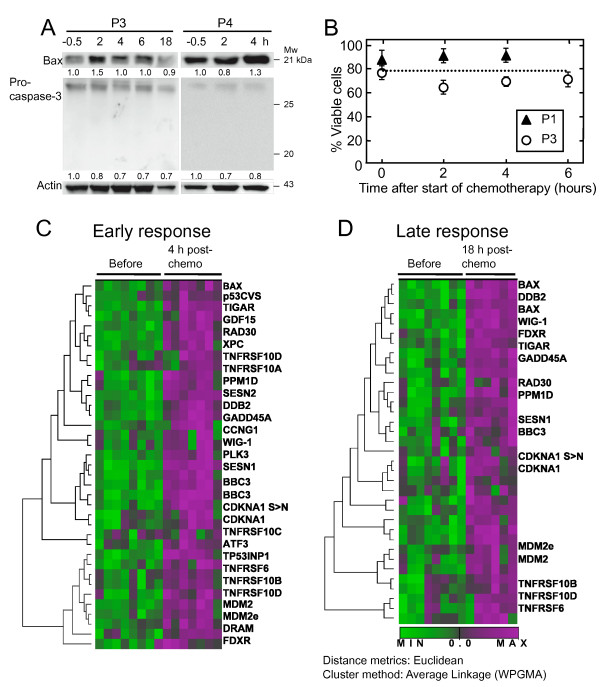
**DNA microarray analysis in AML cells during *in vivo *chemotherapy**. AML blast viability was investigated in four patients sampled during *in vivo *chemotherapy. The expression of apoptosis related proteins were detected by Western blotting (P3, P4) (**A**). Expression of the pro-apoptotic protein BAX increased during therapy, suggesting initiation of cell death. However, no cleavage of the executioner protease pro-caspase-3 could be detected and this is in agreement with the flowcytometric analysis in (B). Apoptosis was measured using flowcytometric detection of Annexin-V FITC and propidium iodide staining (P1, P3) as described in Methods. No signs of apoptosis could be detected in the cells during the first six hours of therapy in either patient (**B**). Similar viability (~80%) was present at 18 h determined by analysis of nuclear morphology and cell scattering (data not shown). Early changes in gene expression of p53-associated genes were detected already 2–4 hours following induction therapy (**C**). The profiles of 27 of the 31 p53 associated genes are shown. Late response gene expression of p53-inducible genes was detected 18–24 hours after treatment induction (**D**). For abbreviations see Additional File 2. The dendrogram and heat maps show a Eucledian two-way cluster analysis based on the most consistently upregulated genes. Thus, genes that cluster together have a similar expression profile as a response to induction therapy. In the diagram the relative mRNA levels in the blasts before therapy are colored in green, and upregulated genes following standard chemotherapy treatment are shown in violet according to the color scale below.

In this study we examined seven AML patients before (-30 min), at 2–4 h and at 18–24 h after start of first day standard induction therapy (for patient characteristics, see Additional File 1) [26], consisting of a 30 min infusion of anthracycline and a 24 h continuous infusion of cytarabine started concomitantly. According to our local guidelines we preferred use of idarubicin instead of daunorubicin in patients below 60 years of age. There is limited documentation that idarubicin has therapeutic advantages over daunorubicin, and we regard these anthracyclines as equal in therapy of AML. It has previously been reported that apoptotic cells are absent in bone marrow and peripheral blood during intensive chemotherapy of AML for at least 24 h, even if the cell numbers in peripheral circulation start to decline [27,28]. Based on these reports and our own observations (Figure 1) we assumed that AML blasts from patients early after chemotherapy represent pre-apoptotic AML cells. Two AML patients outside this gene expression analysis demonstrated unaltered white blood cell (WBC) and lymphocyte counts or a 50% reduction in WBC and 20% reduction in lymphocytes at 24 h, respectively. Patient 1 and 4 (Additional File 1) had 12 and 23% reduction in WBC, respectively, 24 h after start of chemotherapy. We therefore conclude that the fraction of cancer cells at 4 and 18 h after start of chemotherapy was nearly similar. Western blot analysis of two samples (P3 and P4) detected a treatment related induction of BAX protein but no cleavage product of procaspase-3 was observed 2 to 18 h after start of therapy (Figure 1A). As expected no apoptosis was observed with flow cytometric analysis of annexin V FITC and propidium iodide staining (P1, P3) during the first 6 hours of treatment (Figure 1B), and examination of AML blasts for cell shrinkage or changes in nuclear morphology within 24 h after start of treatment revealed no signs of apoptosis (data not shown).

### FLT3 mutation indicated low survival and a blunted therapy-induced gene expression

FLT3 length mutation/internal tandem mutation (ITD) is strongly associated with disease relapse and low overall survival [14]. We compared AML patients (Additional File 1) with wild type (2/7, mean survival 49 months, 28 and >69 months respectively) and ITD FLT3 (5/7, mean survival 8 months, range 0–28), and found that at pretreatment only 11 genes differed with false discovery rate of zero (0). This included a 12-fold increase in HOXA5 and a 24-fold increase in azurocidin. Four hours after start of chemotherapy, 162 genes were modulated with false discovery rate of zero (0), but only 43 of these genes were upregulated. This suggests that treated patients with wild type FLT3 activate genes involved in gene transcription more vividly compared to patients with FLT3 ITD-mutation. The low number of patients and the heterogeneity in AML implicate that these observations need to be explored in a larger cohort of AML patients.

### Identification of p53-associated gene expression in AML cells after induction therapy with anthracycline and cytarabine

Samples from all treated patients were analyzed using Agilent 44 k oligonucleotide microarrays and comparing gene expression before and after induction therapy with anthracycline and cytarabine. 113 upregulated genes (23 of unknown function) at early time points (2 – 4 hours) and 108 genes at late time points (18 – 24 hours) performed a minimum fold change of 1.6 and false discovery rate (FDR) below 11%. Downregulation was likewise observed for 38 genes at early and 17 genes at late time points after treatment. Several of the highly overexpressed genes did not have known function and performing BLAST analysis revealed that several of them contained Alu-like sequences. The downregulated genes showed low fold changes ranging from 1.6 to 2.0. The 113 upregulated genes (1.7 – 8.1 fold) in AML blasts 2–4 h after induction chemotherapy included 31 genes related to the tumor suppressor p53 (Additional File 2). The mRNA levels of most of these genes except for BAX decreased at the end of the first cytarabine infusion, 18–24 h after start of treatment. The dendrogram and heat map presentations demonstrate the consistently upregulated p53 related genes during treatment (Figure 1C and 1D). Genes induced during the first hours of treatment also included the proliferative constellation of ATF3 (activating transcription factor 3), CREB1 (cAMP responsive element binding protein 1), PCNA (proliferating cell nuclear antigen) and EP400 (E1A binding protein p400). An 8-fold overexpression of the NOTCH modifier lunatic fringe (Drosophila) homolog, LNGF, together with the NOTCH homolog 2 N-terminal like protein, NOTCH2NL, was also observed.

Clinically relevant concentrations of anthracyclines induce apoptosis within hours after start of treatment *ex vivo *[3]. AML cells obtained from one patient (P8) prior to chemotherapy were also treated *ex vivo *with 8.0 μM daunorubicin for 4 h in order to compare with *in vivo *changes of gene expression. Samples from the *ex vivo *experiment were also analyzed using Agilent cDNA microarrays and the results subjected to the same statistical analyses as described in Methods. The p53 associated genes obtained from the *in vivo *and *ex vivo *study are summarized in Additional File 2, predominantly supporting the *in vivo *treatment observation.

### p53 directs pre-apoptotic events through the FAS/TRAIL and the mitochondrial apoptotic pathway

**Figure 2:**
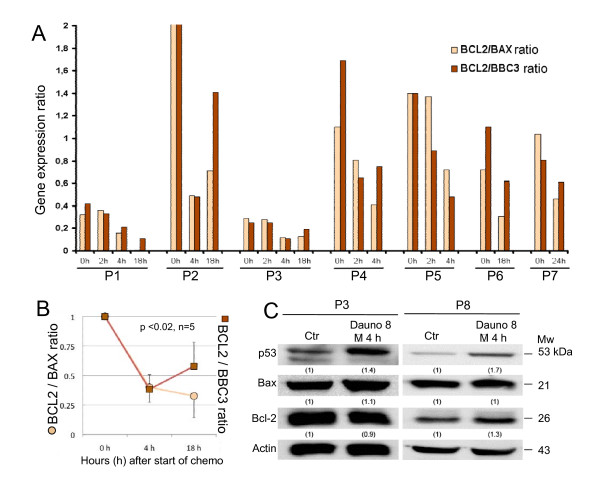
**Expression of apoptosis modulators BCL-2, BAX and BBC3 during chemotherapy**. The ratio of BCL2/BAX or BCL2/BBC3 mRNA was determined after Agilent Human Whole Genome Oligo Microarray analysis (**A**). Ratios were observed to decrease in the first hours post induction treatment, indicating an increase in pro-apoptotic gene expression. Ratios varied greatly between patients and low baseline ratios were associated with better response to treatment (Additional File 1). Ratios were normalized and mean presented with standard deviation (error bars) (**B**), indicating early pro-apoptotic alteration in BCL2/BAX mRNA. Two patients (P3 and P8) were treated with anthracycline *ex vivo *and protein expression analyzed using Western blots (**C**). The *ex vivo *response to anthracycline included increased stabilization of p53 followed by regulation of p53 target gene expression in concordance with *in vivo *observations.

Altogether five tumor necrosis factor-related receptor genes were modulated 2–4 h after induction therapy (Additional File 2). We also investigated the BCL2/BAX and BCL2/PUMA ratios as apoptotic responders to treatment at the different time points for each of the patients, and found a significant increase in gene expression of the apoptosis facilitators PUMA and BAX (Additional File 2) and a decrease in the BCL2/BAX ratio as well as BCL2/PUMA for most of the AML samples (0 versus 4 h p-values < 0.0004, 0 versus 18 h p-values < 0.01, 4 versus 18 h p-values 0.5 and 0.2, respectively) (Figure 2A,B). The mRNA profile of three other pro-apoptotic mediators BAD, BAK1 and BIM did not change significantly during the first hours, but the level of gene expression varied across patients. There was no significant alteration of MCL-1 mRNA during chemotherapy. Two patients (P3 and P8) were selected for *ex vivo *treatment experiments and determination of protein expression of the p53-targeted genes BAX and BCL-2, and showed an appropriate induction of these p53 regulated gene products (Figure 2C) in concordance with mRNA expression. BAX mRNA was not induced in P8 during the first 4 h of treatment, and several anti-apoptotic genes increased. This is also reflected in the protein expression of BAX along with an increase of BCL-2 protein (Figure 2C). The lowest BCL-2/BAX ratio was observed in patient 1 and 3, and these patients had the longest observed survival in this limited cohort of patients (Additional File 1).

### Genes involved in chemoresistance and cell-cell contact are expressed 18 h after start of chemotherapy

**Figure 3:**
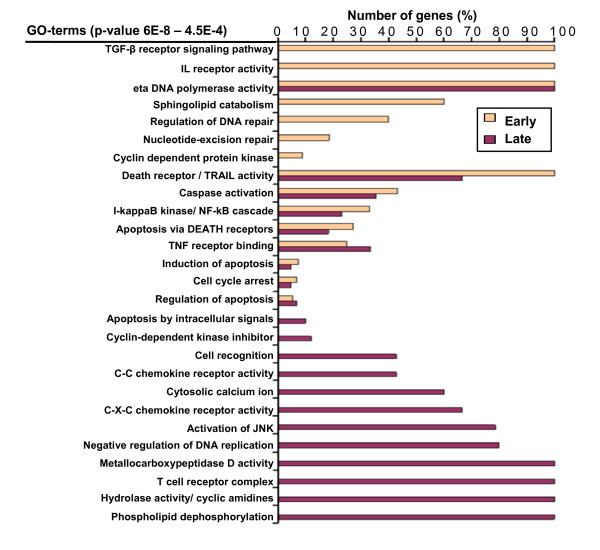
**Gene ontology analysis of chemotherapy induced genes**. Gene ontology (GO) annotations associated with overexpressed genes that significantly discriminate between Early (2–4 h) and Late (18–24 h) responses were determined using the Source database http://source.stanford.edu and iterative group analysis (iGA) [22]. All overexpressed genes in the array were analyzed to determine gene products associated with any GO-term. The significant annotations consisting of genes found to be significantly up- or downregulated for early and late response were selected by use of 100 permutations. The threshold for significant GO annotations was set to *p*-value 4.5E-4. Number of genes (%), horizontal axis, represents the proportion of the identified genes belonging to each GO term that significantly changed their expression at early or late timepoints after start of chemotherapy.

To allow an overview of genes modulated by chemotherapy, we searched for functional gene ontology (GO) annotations associated with the upregulated genes that discriminate early and late response to the drugs (Figure 3, Additional File 3). In total, 42 biological functions were significantly associated with *in vivo *response of treatment. Fifteen GO terms delineated coherent functions related to apoptosis (e.g. DNA damage, regulation of cell cycle, regulation of apoptosis by TNF receptors and mitochondrial signals). In contrast, the cells collected after approximately 24 h of chemotherapy demonstrated an increasing number of genes related to cell-cell contact (Figure 3); this included subunits of the T cell receptor complex and chondroitin sulphate-containing proteins involved in cell recognition. Within 2–4 h NOTCHL2 and LUNATIC were induced, as part of pathways that may be related to the later T-cell receptor gene induction. Both NOTCH [29] and the myotubularin (MTM) family may be involved in chemoresistance [30]. Likewise, chemokine receptors CXCR4 and CX3CR1 were upregulated in this late phase after start of chemotherapy. Integrin binding and CXCR4 chemokine receptor activation are important for migration of CD34+ hematopoietic progenitors and AML cells to marrow stroma cells [31], and leukemia cells may utilize CXCR4 to access niches that are normally restricted to progenitor cells, and thereby home into a microenvironment that favours their growth and survival [31,32]. Together, this may indicate hitherto unknown interactions between leukemic cells and host compartments during chemotherapy.

## Discussion

Differentially overexpressed genes at 2–4 h included p53-induced genes related to oxidative stress, cell cycle arrest, DNA repair, autophagy and apoptosis (Figure 1C, Additional File 2). Although moderate, the early upregulation of the p53 target gene DRAM (damage-regulated autophagy modulator), encoding a lysosomal protein, stands out as special [33,34]. It has been reported that autophagy may delay the DNA damage response and apoptotic death in breast cancer cells [35] and this duality indicates a highly complex regulation of cell fate post therapy.

In addition to the p53-induced genes with putative anti- and pro-survival function (Additional File 2), several novel features were observed. Upregulation of cytidine deaminases (cyclic amidines) represents a significant mechanism for inactivation of cytarabine and thereby confers therapy resistance in AML (Figure 3 and Additional File 3) [36]. Another class of genes involved in cisplatin resistance in solid cancers is the myotubularin family, some of them encoding proteins with dual phosphatase activity [30,37]. Several genes indicate a tumor-host modulation early during chemotherapy. This includes the upregulation of chemokine receptors like CXCR4, whose expression levels have been proposed of major prognostic impact in acute myeloid leukemia [31].

The BCL2/BAX ratio as well as BCL2/BBC3 (PUMA) ratio decreased at early time points in all samples analyzed (Figure 2A, B). Interestingly, the baseline ratios were particularly high for two of the patients with poor outcome as has also been reported for other patients with adverse prognosis [38], while the two patients with the lowest baseline ratios responded best to first course induction therapy (Figure 2A and Additional File 1). Induction of BAX was accompanied by increase of BBC3 mRNA, a proposed link between p53 and BAX [39], which in turn connects TRAIL receptors with mitochondrial apoptosis [40]. The pro-apoptotic genes, along with p53-inducible death domain (PIDD/LRDD) [41] were expressed at 18–24 h, but was not reflected in cleaved procaspase-3 as verified by Western blotting (Figure 1A).

Studies by others have suggested that apoptotic AML blasts will not be detected in peripheral blood samples during chemotherapy [27,28]. Dedicated phagocytes or neighboring cells are presumably clearing these apoptotic AML cells from circulation through receptors and adaptor molecules that can link apoptotic cells to phagocytes (reviewed in [42,43]). Our patients experienced no clinical symptoms of tumor lysis, reflecting an intact absorbance of apoptotic cells in the patient undergoing chemotherapy. This observation was consistent with absence of apoptosis by annexin V/propidium iodide analysis or procaspase-3 cleavage (Figure 1). Routinely, nuclear morphology was examined after density gradient centrifugation of mononuclear cells, and nuclei were not observed fragmented or condensed (data not shown). Our previous reports of pre-apoptotic BAX induction in vitro [44], and the lack of cleaved caspase substrate proteins in AML patient samples support the conclusion that the early phase after chemotherapy represents a physiological window to examine pre-apoptotic gene modulation *in vivo*. We observed no induction of genes that are involved in the classical clearance of apoptotic cells in the circulating AML blasts [42,43] except for an induction of the extracellular molecule MFGE8 (milk fat globule-EGF factor 8 protein) (data not shown). MFGE8 is like annexin V capable of binding to phosphatidylserine (PS) and facilitates engulfment by bridging PS on the apoptotic cell with macrophages [45]. Another indication of apoptotic cell clearance was a 9-fold increase in mRNA expression of GalNAc4S-6ST (N-acetylgalactosamine 4-sulfate 6-O-sulfotransferase), and a high level of this molecule has been detected on apoptotic human peripheral blood lymphocytes [43]. Furthermore, several receptors that are expressed on monocytic/macrophage lineage cells were upregulated, probably related to chemotherapy induced differentiation of the leukemic cells [46]. We hypothesize that lack of classical "eat-me" signals of the pre-apoptotic cells in this study is caused by the nature of chemotherapy induced cell death and cell phenotype.

We observed increased expression of T-cell receptor complex components after 18–24 h of therapy (Figure 3). AML cell number in peripheral blood was rapidly declining after chemotherapy and partly followed by a decrease in lymphocytes, not sufficient to explain the increase in T-cell receptor related genes. The chemotherapy induced expression of T-cell receptor complex genes could reflect aberrant gene expression of leukemic blasts, mirroring the flexible pattern of gene expression observed in pluripotent hematopoietic stem cells [47].

## Conclusion

In conclusion, this study of gene expression of AML blasts *in vivo *following start of induction chemotherapy confirms the vivid response to therapeutic DNA damage observed in experimental systems. The most prominent genes that were upregulated immediately after chemotherapy are pivotal determinants of apoptosis regulation *in vitro *(Additional File 4). More striking is the upregulation of genes potentially involved in interaction between AML blasts and the host microenvironment, supporting the hypothesis that the host response in chemotherapy is crucial for persistent remission [48]. The observations presented here provide us with a more nuanced picture of leukemic cell demise after intensive chemotherapy *in vivo*, and motivate for an expanded patient study to search for possible therapy response biomarkers.

## Competing interests

The authors declare that they have no competing interests.

## Authors' contributions

AMØ designed the study, performed experiments, analyzed data, and wrote the manuscript. NA performed experiments, analyzed data, wrote the manuscript. THB and LS analyzed data and reviewed the manuscript. IJ designed the bioinformatics study and reviewed the manuscript. ØB provided biological material, designed the study and reviewed the manuscript. KHK designed the study and wrote the manuscript. BTG designed the study, provided biological samples, analyzed data, and wrote the manuscript.

## Pre-publication history

The pre-publication history for this paper can be accessed here:

http://www.biomedcentral.com/1471-2407/9/77/prepub

## Supplementary Material

Additional file 1 **Clinical and biological characteristics of acute myeloid leukemia patients. **Table displaying clinical and biological characteristics of acute myeloid leukemia patients Click here for file

Additional file 2 **p53-interacting genes expressed in AML blasts following treatment *in vivo *with anthracycline and cytarabine and *ex vivo *with anthracycline.** Average fold increase of gene expressions in isolated blasts from 7 AML patients 2–4 h (early response) and 18–24 h (late response) following treatment in vivo with anthracyclines according to Oligo DNA arrays, cDNA arrays and TaqMan Low Density Array (T-LDA). Click here for file

Additional file 3 **Permuting relationship between GO-terms and genes induced at late response following *in vivo *chemotherapy.** The iterative group analysis was used to analyze whether gene products associated with any term in gene ontology (GO) were over-represented among those found to be significantly regulated for early and late response. Source http://source.stanford.edu. False discovery rates for the list of reported GO-terms were estimated by performing 100 permutations. Click here for file

Additional file 4 **Early gene expression signature of chemotherapy treated AML *in vivo*.** The gene expressions of p53-associated genes implicated in the response to oxidative stress, cell cycle arrest, DNA repair, autophagy and apoptosis using standard anthracycline and cytarabine-based chemotherapy are shown. Red colored receptors and nodes represent upregulated genes observed within the first 24 hours of chemotherapy *in vivo*. Click here for file
