# Supplementary material for: Genes of cell-cell interactions, chemotherapy detoxification and apoptosis are induced during chemotherapy of acute myeloid leukemia
Source: BMC Cancer. 2009 Mar 5;9:77. doi: 10.1186/1471-2407-9-77 (PMC2673224; doi:10.1186/1471-2407-9-77)
Supplement: Additional file 1 — Clinical and biological characteristics of acute myeloid leukemia patients. Table displaying clinical and biological characteristics of acute myeloid leukemia patients [file 1471-2407-9-77-S1.doc]

**Additional File 1. Clinical and biological characteristics of acute myeloid leukemia patients**

___________________________________________________________________________________________________________________________

**Membrane molecules** Time to Remission after Survival

Patients Sex Age FAB CD13 CD14 CD15 CD33 CD34 Karyotype FLT3 NPM1 WBC^*^ Chemo Cytopenia^#^ 1^th^ induction (months) ________________________________________________________________________________________________________________________________________

P1 M 56 M1^§^ + - - + + inv(1),t(2;10),t(9;22) WT ND 6 IC 5 + >69

P2 M 30 M3 + - - + + t(15;17) LM WT 29 IC 3 ND 0

P3 M 61 M4/M5 + - + + - Normal LM M 71 IC 6 + 20

P4 M 64 M4/M5 + - - + - Normal LM M 135 I ND - 0

P5 M 70 M1 + - - - + Normal WT ND 2 DC 9 - 28

P6 F 63 M4 + - - + + Normal LM WT 86 DC 5 - 6

P7 M 29 M4 + - - + + Normal LM M 17 IC 7 - 12

P8 F 38 M5a - - + + - t(9;11) D835 WT 286 (IC) 7 + >88

**Note:** FAB, French-American-British classification of acute myeloid leukemia. Membrane molecules were determined by flow cytometry and scored as positive if more than 20% of the leukemic blast population was positive, see Øyan *et al.* 2005 for details, [18]. ^*^WBC, white blood cells (x10^9^/L) at diagnosis before therapy. ^#^Time to cytopenia indicates days before WBC counts were below 0.5x10^9^/L. FLT3 and NPM1/nucleophosmin 1 mutational status detected by PCR technique as previously described. LM, FLT3 length mutation; M, NPM1 mutation; ND, not determined, Chemo, Chemotherapy consisted of three days with idarubicin (I) or daunorubicin (D), and concomitantly start up with cytarabine for seven days (C). Anthracycline was infused over 30 min and cytarabine for 24 h as an continuous infusion; ^§^Patient P1 experienced a four months period with anemia and malady before the diagnosis of AML was confirmed with more than three chromosomal aberrations including inv(1)(p22,p34),t(2;10)(q33;q22),t(9;22)(q34;q11). The finding of high platelet counts 1290x10^9^/L (normal range 145-348x10^9^/L) was examined with analysis of AML blasts for megakaryocytic/platelet marker CD49, but was negative. Presence of t(9;22) and high platelets could be consistent with the differential diagnosis chronic myeloid leukemia in blast crisis, but this translocation is also found infrequently in AML. Cytology showed immature Sudan negative blasts, concluding on FAB M1. The patient has been in cytogenetic remission after receiving long-term maintenance therapy with imatinib; Patient P4 received reduced dose idarubicin (I) as cytoreductive treatment shortly after admittance in an attempt to reduce disseminated intravascular coagulation and avoid progress of organ failure. The patient died within the first 24 h; P8 received treatment IC and allogeneic bone marrow transplant, but in this study the sample was collected before therapy and treated *ex vivo* with daunorubicin. Remission after induction was defined as less than 5% myeloid blasts in bone marrow aspirate determined by microscopy two weeks after start of chemotherapy.
