# Supplementary material for: Genes of cell-cell interactions, chemotherapy detoxification and apoptosis are induced during chemotherapy of acute myeloid leukemia
Source: BMC Cancer. 2009 Mar 5;9:77. doi: 10.1186/1471-2407-9-77 (PMC2673224; doi:10.1186/1471-2407-9-77)
Supplement: Additional file 2 — p53-interacting genes expressed in AML blasts following treatment in vivo with anthracycline and cytarabine and ex vivo with anthracycline. Average fold increase of gene expressions in isolated blasts from 7 AML patients 2–4 h (early response) and 18–24 h (late response) following treatment in vivo with anthracyclines according to Oligo DNA arrays, cDNA arrays and TaqMan Low Density Array (T-LDA). [file 1471-2407-9-77-S2.doc]

**Additional File 2. p53-interacting genes expressed in AML blasts following treatment *in vivo* with anthracycline and cytarabine and *ex vivo* with anthracycline**

_______________________________________________________________________________________________________________________________________

In vivo Ex vivo P8

__________________________________ __________

Oligo Array cDNA Array LDA cDNA array

Gene Gene name GenBank* Early Late Early Early Early

_______________________________________________________________________________________________________________________________________

MDM2 p53-binding protein MDM2 NM_002392 8.1 3.6 4.1 - 2.4

FDXR Ferredoxin reductase mitochondrial protein NM_004110 5.1 3.2 - 7.2 -

p21/Cip1 Cyclin-dependent kinase inhibitor 1A NM_000389 4.6 3.9 5.1 5.7 2.1

CIP1/WAF1 Cyclin-dependent kinase (CIP1/WAF1) S->R mutation L47232 4.2 5.1 5.4 - 2.0

TNFRSF10D Tumor necrosis factor receptor superfamily, 10D Decoy-R2 NM_003840 3.6 2.1 - 4.2 -

GDF15 Growth differentiation factor 15 NM_004864 3.5 2.0 - 2.4 -

BBC3 BCL2 binding component 3 NM_014417 3.3 2.6 2.3 4.0 1.5

GADD45A Growth arrest and DNA-damage-inducible, alpha NM_001924 3.3 2.3 3.5 3.9 2.6

PLK3 Polo-like kinase 3 NM_004073 3.2 1.6 2.2 4.9 1.3

TIGAR TP53-induced glycolysis and apoptosis regulator NM_020375 3.1 1.8 1.3 - 1.6

SESN2 Hypoxia induced gene 95 NM_031459 3.1 1.6 - 3.5 -

PLK2 Polo-like kinase 2 NM_006622 2.9 2.2 - 12 -

TNFRSF10B Apoptosis inducing receptor TRAIL-R2 NM_003842 2.8 2.6 2.7 3.3 1.5

SESN1 p53 regulated PA26 nuclear protein I_957946 2.6 2.4 2.6 - 2.1

DDB2 Damage-specific DNA binding protein 2, 48kDa NM_000107 2.4 2.9 2.6 - 1.8

PPM1D Protein phosphatase Wip1 NM_003620 2.3 1.8 2.2 - -

TNFRSF6 Tumor necrosis factor receptor superfamily, member 6 NM_000043 2.3 2.0 2.2 2.3 -

P53CSV p53-inducible cell-survival factor NM_016399 2.2 1.7 - - 2.0

TP53INP1 Tumor protein p53 inducible nuclear protein 1 NM_033285 2.0 1.6 - - -

RAD23B RAD23 homolog B BC020973 1.9 - - - -

ATF3 Activating transcription factor 3 BC006322 1.9 1.8 1.6 - 1.4

XPC Xeroderma pigmentosum, C NM_004628 1.9 1.7 1.7 - -

TNFRSF10C Tumor necrosis factor receptor superfamily, 10C Decoy-R1 NM_003841 1.8 1.7 - - -

BAX BCL2-associated X protein β NM_004324 1.8 1.9 - 2.2 -

RRM2B Ribonucleotide reductase M2 B (TP53 inducible) NM_015713 1.8 - 1.9 - 1.6

CCNG Cyclin G1 NM_004060 1.8 1.6 1.8 2.0 1.4

WIG1 p53 target zinc finger protein AK022358 1.8 2.5 - - -

ACTA2 Actin, alpha 2 NM_001613 1.8 2.4 - - -

LRDD PIDD, p53-induced protein with a death domain NM_145886 - 1.9 - - -

DRAM Damage-regulated autophagy modulator NM_018370 1.7 - - - - TNFRSF10A Tumor necrosis factor receptor superfamily, 10A NM_003844 1.6 - - 1.9 -

_________________________________________________________________________________________________________________________________________

Average fold increase of gene expressions in isolated blasts from 7 AML patients 2-4 h (early response) and 18-24 h (late response) following treatment in vivo with anthracyclines according to Oligo DNA arrays, cDNA arrays and TaqMan Low Density Array (T-LDA). - not detected. * GenBank numbers DNA oligo arrays
