# Supplementary figures and images for: Genes of cell-cell interactions, chemotherapy detoxification and apoptosis are induced during chemotherapy of acute myeloid leukemia
Source: BMC Cancer. 2009 Mar 5;9:77. doi: 10.1186/1471-2407-9-77 (PMC2673224; doi:10.1186/1471-2407-9-77)

## Slide 1
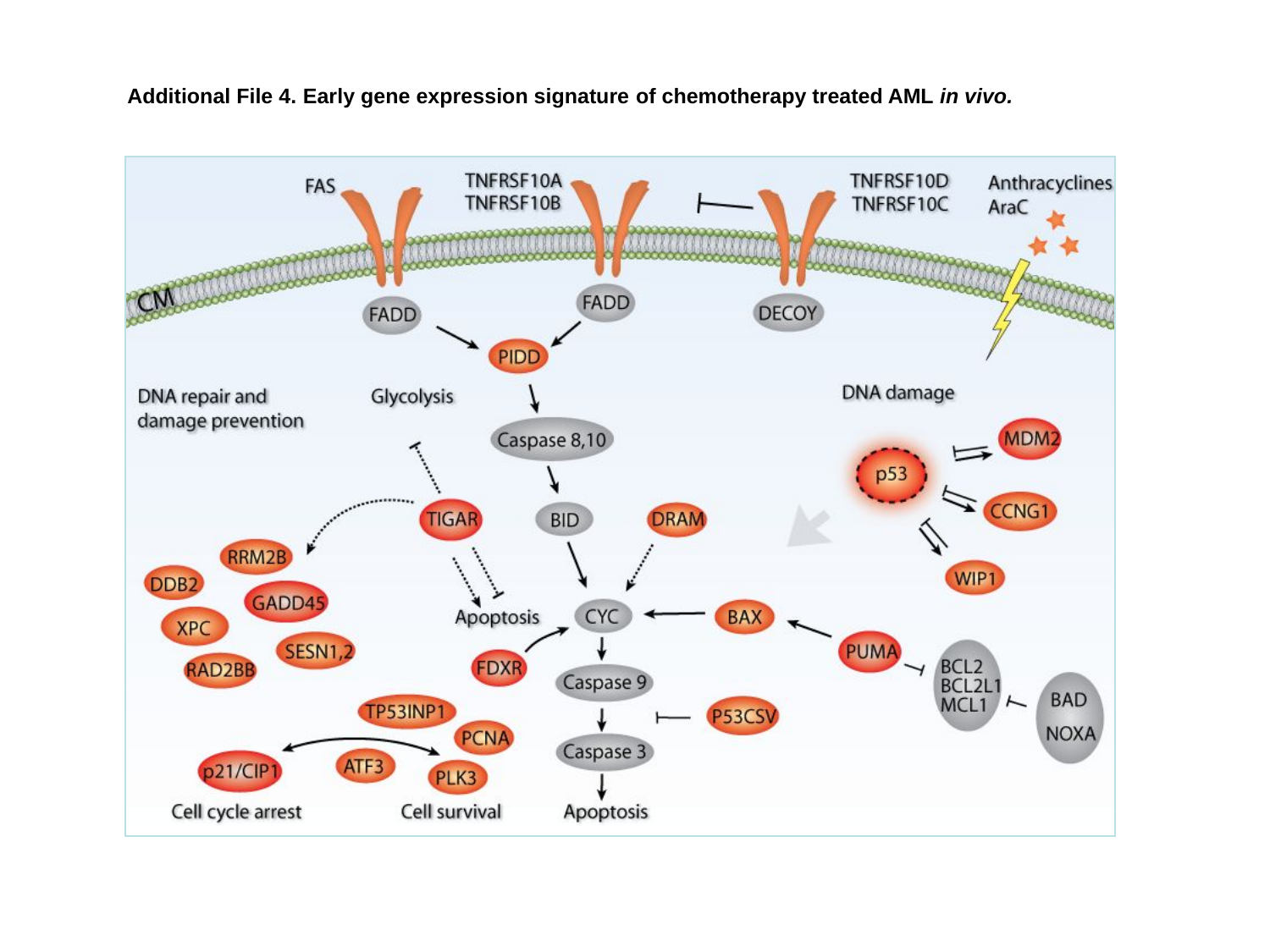

Additional File 4. Early gene expression signature of chemotherapy treated AML in vivo.

Supplement: Additional file 4 — Early gene expression signature of chemotherapy treated AML in vivo. The gene expressions of p53-associated genes implicated in the response to oxidative stress, cell cycle arrest, DNA repair, autophagy and apoptosis using standard anthracycline and cytarabine-based chemotherapy are shown. Red colored receptors and nodes represent upregulated genes observed within the first 24 hours of chemotherapy in vivo. [file 1471-2407-9-77-S4.ppt]
